# The Efficacy and Safety of Transurethral 2 μm Laser Bladder Lesion Mucosal En Bloc Resection in the Treatment of Cystitis Glandularis

**DOI:** 10.3389/fmed.2022.840378

**Published:** 2022-03-17

**Authors:** Changyuan Zhao, Kexin Wang, Chao Men, Yue Xin, Haibo Xia

**Affiliations:** Department of Urology, Chifeng Cancer Hospital, Chifeng, China

**Keywords:** cystitis glandularis, 2 μm laser, en bloc resection, transurethral, bladder

## Abstract

**Objective:**

To study the safety and feasibility of transurethral bladder lesion mucosal en bloc resection with 2 μm laser for cystitis glandularis.

**Methods:**

From July 2018 to July 2019, 58 patients with cystitis glandularis received surgical treatment were selected. All patients in this study were randomly divided into experimental group (transurethral 2 μm laser bladder lesion mucosal en bloc resection) and control group (traditional transurethral resection of bladder lesion mucosal). By analyzing the perioperative and follow-up clinical data of these two operation procedures, we discuss the efficacy and safety of transurethral 2 μm laser bladder lesion mucosal en bloc resection in the treatment of cystitis glandularis.

**Results:**

Patients of two groups received operation successfully without serious complications such as bladder perforation. Compared with the experimental group, the laser treated group hadless bleeding in operation, shorter post-operative catheter indwelling time. These differences were statistically significant. No significant difference existed between two groups in terms of operative time, Bladder flushing time, irritation symptoms of bladder.

**Conclusion:**

Transurethral 2 μm laser bladder lesion mucosal en bloc resection is safe and effective for the treatment of cystitis glandularis, and it is worthy of further clinical application.

## Introduction

Cystitis glandularis is characterized by abnormal proliferation of bladder mucosa caused by multiple factors. In recent years, the incidence of glandular cystitis is gradually increased. The etiology and pathogenesis of glandular cystitis is currently not entirely clear. And related studies have shown that there is a certain association between glandular cystitis and bladder cancer (1, 2). For the treatment of glandular cystitis, some people advocate conservative treatment such as the use of quinolone antibiotics and cyclooxygenase-2 inhibitors (3). There is a view that CG precancerous lesions should be actively treated surgically. There are open surgery methods such as resection of the bladder mucosa after incision of the bladder, partial or total bladder resection, and transurethral surgery for bladder lesions such as transurethral plasma excision or laser resection, etc. Due to minimally invasion of body, transurethral bladder lesion mucosal resection has become the main surgical treatment at present, but it has a high recurrence rate. With the development of scientific and technological progress and renewal of a variety of surgical devices, the clinical treatment way of glandular cystitis has varied nowadays. In this study we explored the new surgical method, transurethral 2 μm laser bladder lesion mucosal en bloc resection, and assessed its efficacy and safety for glandular cystitis treatment (4).

## Materials and Methods

### Clinical Data

**Figure 1:**
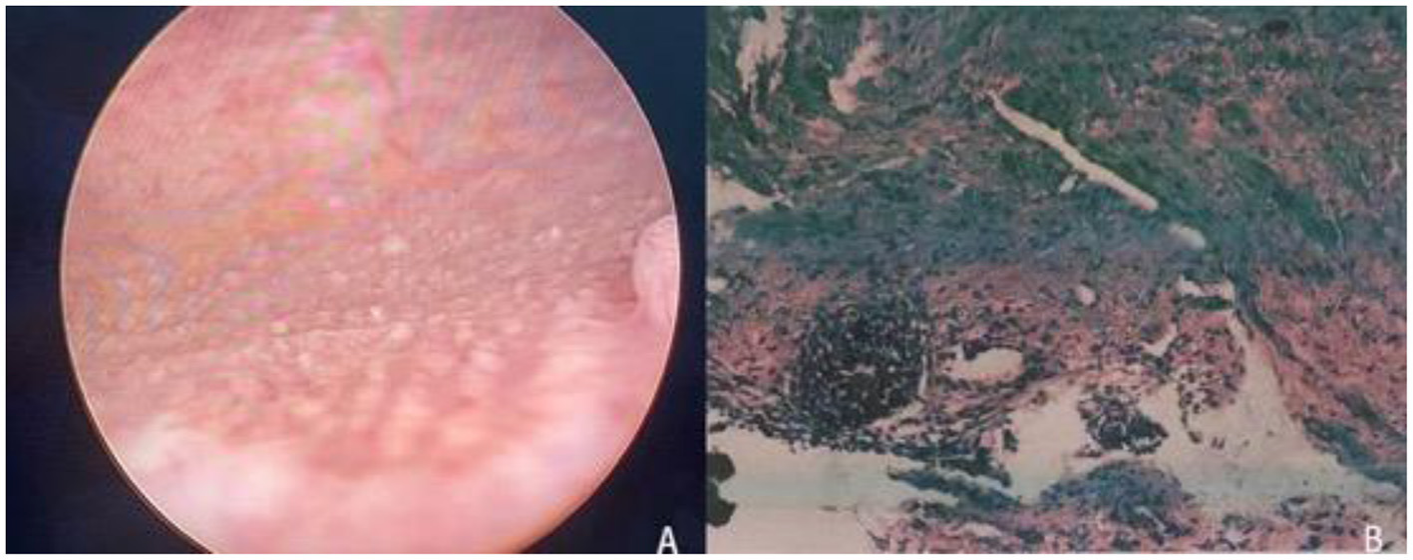
**(A)** Cystoscopy revealed multiple follicular masses in the trigone of the bladder. **(B)** Pathological examination: the submucosa can be seen with adenoid-shaped cell clusters, and the surface of the glandular lumen is covered with normal urothelial cells (H.E × 40).

**Figure 2:**
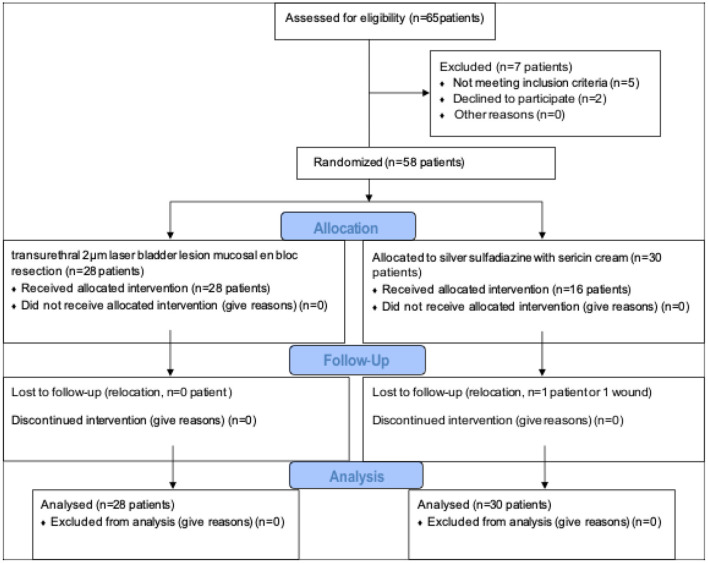
CONSORT flow diagram.

The patients who were diagnosed with cystitis glandularis from 2018.7 to 2019.7 and received surgical treatment were collected. Inclusion criteria: Patients diagnosed with glandular cystitis by pre-operative cystoscopy and pathological (Figure 1). Exclusion criteria: (1) Patients with bladder neck opening obstruction and urethral stricture. (2) Patients who do not want surgery. (3) Patients with contraindications for surgery. Display the results calculated by SPSS, a sample size of 60 patients will give us a power of 0.8 with a α of 0.05. The sample size also takes into account a pre-determined 10% drop rate. Finally, 58 patients with cystitis glandularis received surgical treatment were selected. The enrolled patients were randomly divided into two groups by statistical software: laser group (transurethral 2 μm laser bladder lesion mucosal en bloc resection, 28 cases, 8 males, 20 females,) and TUR group (traditional transurethral resection of bladder lesion mucosal, 30 cases, 6 males, 24 females) (Figure 2). Both groups of patients underwent intravesical instillation therapy received epirubicin once a week for 4 consecutive weeks. The average age was 55.46 ± 6.58 (range 30–67) years old in laser group and 55.67 ± 6.8 (range 28–71) years old in TUR group.

#### General Condition of the Patient

The incidence of glandular cystitis in male patients was lower than female patients in this study (Table 1).

**Table 1:** Baseline characteristics.

**Group**	**Sex**		**Age**
	**Male**	**Female**	
**Laser group**	**8 (28.57%)**	**20 (71.43%)**	**55.46 ± 6.58**
**TUR group**	**6 (25%)**	**24 (75%)**	**54.67 ± 6.8**
	**14 (26.92%)**	**44 (73.08%)**	

#### Clinical Manifestations

Patients with glandular bladder inflammation mainly suffered bladder irritation such as dysuria, urgency, and frequency, and some patients may have hematuria.

### Related Inspections

#### General Inspection

All patients received complete routine examinations of blood, urine, liver and kidney function, electrolytes, coagulation function, and infectious diseases. Patients need to complete the chest X-ray and ECG examination before the operation. Due to the patient's past medical history, personal medical history, age and other related factors, some patients need to complete other related examinations to exclude surgical contraindications.

#### Imaging Examination

##### Urinary System Color Doppler Ultrasound

All patients included in this experiment underwent urinary system color Doppler ultrasound examination before surgery. For male patients, the prostate should also be examined to find out whether the patient has prostate-related lesions.

##### Abdominal CT

CT examination should be executed to check the degree of lesions of the patient's bladder mucosa, and the relationship between diseased mucosa and different layers of tissues of the bladder, and to determine whether there is extra-bladder invasion and the condition of lymph nodes in the pelvis.

##### Intravenous Pyelography

For patients with clinical symptoms such as hematuria or hydronephrosis, intravenous pyelography should be executed to exclude upper urinary tract disease.

##### Cystoscopy Biopsy

All patients included in this experiment underwent cystoscopy before operation and biopsy was proceeded to confirm the diagnosis.

### Surgical Instruments

- Plasma cutting system (SM10).
- Medical RevoLix 2 μm continuous wave laser surgery system (LISA laser products).

### Surgical Method

- Patients of two groups were performed by the same surgeon.

If there are no contraindications to anesthesia, intraspinal anesthesia was used. No obturator nerve block was performed, and normal saline was used as the rinse fluid during the operation.

- Laser group (transurethral 2 μm laser bladder lesion mucosal en bloc resection).

**Figure 3:**
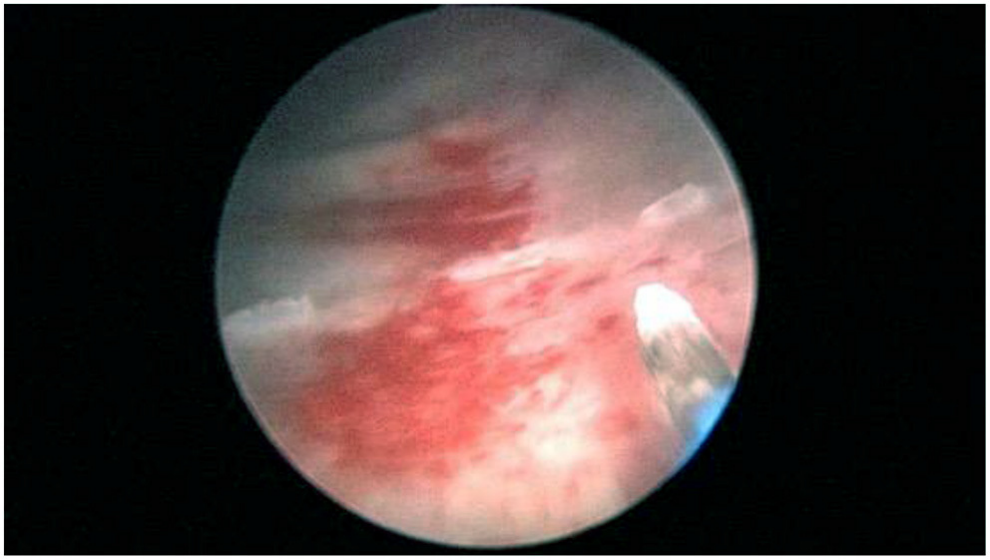
Mark the pre-resection area.

**Figure 4:**
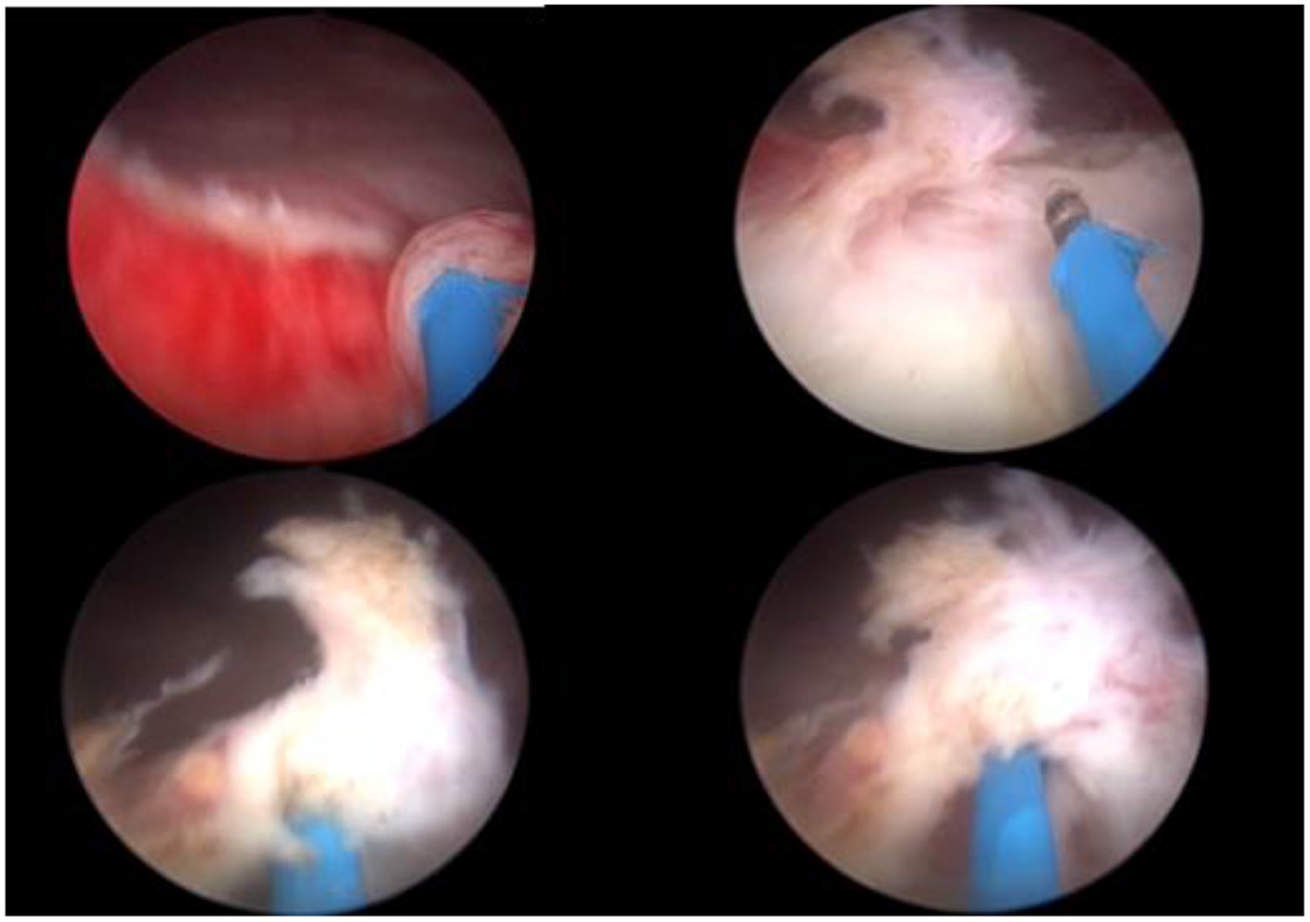
Operation procedure.

**Figure 5:**
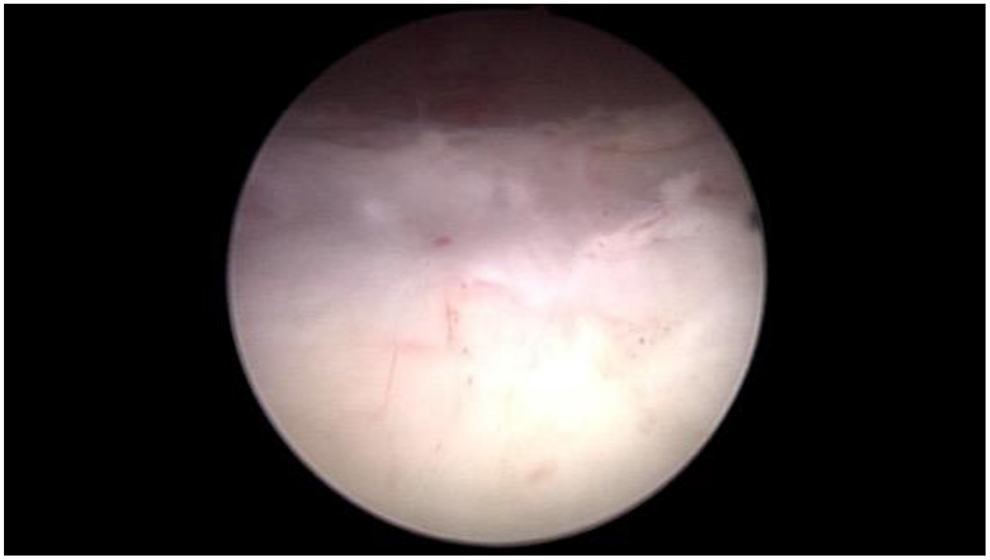
En bloc resection bladder mucosa.

The patient adopted the lithotomy position, fully exposed the perineum, and disinfected the surgical area. To determine a general case such as the position of the lesion, the lesion number, size, range, and clarify the relationship between the diseased mucosa and the ureteral orifice to avoid injury to the ureter during the operation, bladder overall situation was observed with 2 μm laser mirror into the bladder through the urethra. Then a 2 μm laser fiber was inserted and was adjust to be about 1–2 cm beyond the lens of the laser mirror. Use a 2 μm laser to mark the pre-resection area in the normal mucosa at a distance of 0.5–1.0 cm from the edge of the lesion (Figure 3). To reduce intraoperative bleeding, the blood vessels around the lesion could be cauterized with laser. A 2 μm laser fiber was inserted under the bladder mucosa, and bladder mucosa was lifted to maintain a certain tension with the bladder muscle layer, and then the bladder mucosa was cut and separated partly from muscle layer with a certain gap. Blunt peeling was performed along this gap with laser fiber and laser mirror. If the muscle fibers between the diseased mucosa and the bladder wall cannot be broken, using a laser to cut it (Figure 4). En bloc resection bladder mucosa with this method. After flushing out the diseased tissue with an irrigator, laser was used to stop bleeding on the wound (Figure 5). All patients underwent continuous bladder irrigation and catheterization after confirming that there was no bleeding.

- TUR group (traditional transurethral resection of bladder lesion mucosal).

The patient adopts the lithotomy position, fully exposes the perineum, and disinfects the surgical area. Use plasma electrodes to excise the diseased mucosa from front to back, with the depth of excision reaching the superficial muscle layer.

### Data Collection

The perioperative and post-operative follow-up indicators of all patients were recorded. And the patient's operation time, the amount of bleeding during the operation, and the difference in hemoglobin (HGB) values before and after the operation were collected. The occurrence of bladder perforation and obturator reflex during the operation was recorded. And the duration of continuous bladder irrigation, catheter indwelling time and hospitalization days of all patients after surgery were collected and recorded in detail. Regular follow-up after operation was executed to determine whether the patient's various clinical symptoms before operation have improved.

### Treatment Effect Evaluation

① Cured: clinical symptoms disappear completely, no obvious abnormalities after reexamination. ② Symptom relief: the patient's symptoms are better than before, and occasionally symptoms mentioned above appear. ③ Invalid or recurrence: symptoms are not significantly improved or symptoms reappear after a period of time after improvement. Cystoscopy revealed abnormal mucosal changes at the original lesion site again, and the pathology results after biopsy showed glandular cystitis again. ④ Effective: Cured and symptom relief mentioned above.

### Statistical Methods

All data were presented as mean ± SD. Student's *t*-test was used. The data were analyzed statistically using the SPSS 21.0. And *P*-value < 0.05 was considered statistically significant.

## Results

### General Data

Laser group: 28, 8 males, 20 females, male to female ratio: 1:2.5. The average age was 55.46 ± 6.58 (range 30–67) years old. TUR group: 30, 6 males, 24 females, male to female ratio: 1:4. The average age was 55.67 ± 6.8 (range 28–71) years old. There were no significant differences between laser group and TUR group in average age and gender (*P* > 0.05; Table 1).

### Perioperative Indicators

The average operation time of laser group was 23.82 ± 5.19 (range 15–35) min. The post-operative consecutive irrigation times was 1 day, and the mean catheter duration was 4.07 ± 0.77 days in laser group. The average of post-operative hospital stays of laser treated patients was 5.07 ± 0.77 days. As for TUR group, the average operation time was 23.77 ± 5.15 (range 15–35) min. The post-operative irrigation times was 1 day, and the mean catheter duration was 4.00 ± 0.64 days, and the mean post-operative hospital stays was 5.00 ± 0.64 days. There was no significant difference of these indicators between laser group and TUR group (*P* > 0.05).

In experimental group, intraoperative blood loss was 11.89 ± 1.64 ml, and the difference value of hemoglobin drop before and after operation was 4.61 ± 1.10 g/L. As for TUR group, intraoperative blood loss was 23.47 ± 1.85 ml, and the difference value of hemoglobin drop before and after operation was 8.30 ± 1.26 g/L. There was significant difference of these indicators between laser group and TUR group (*P* < 0.05; Table 2).

**Table 2:** Perioperative indicators.

**Group**	**Operation time (min)**	**Post-operative irrigation (day)**	**Catheter time (day)**	**Post-operative hospital (day)**	**Post-operative bleeding (ml)**	**HGB (ml)**
Laser group	23.82 ± 5.19	1	4.07 ± 0.77	5.07 ± 0.77	11.89 ± 1.64 ml	4.61 ± 1.10 g/L
TUR group	23.77 ± 5.15	1	4.00 ± 0.64	5.00 ± 0.64	23.47 ± 1.85 ml	8.30 ± 1.26 g/L
T	0.04		0.385	0.385	−25.12	−11.83
P	0.968		0.701	0.701	<0.001	<0.001

### Post-operative Follow-Up

With 3 cases in each group having symptoms of cystitis glandularis such as irritation symptoms of bladder after the operation, there was no significant difference between the two groups (*P* = 0.631). Follow-up time ranges from 3 to 12 months. During this period, in laser group, 15 cases were cured, 10 cases improved, 3 cases relapsed, and the effective rate and recurrence rate was 89.29 and 10.71%, respectively. As for TUR group, 20 cases were cured, 7 cases improved, 3 cases relapsed, with the effective rate and recurrence rate being 90 and 10%, respectively. There was no statistically significant difference in the recurrence rate using the Fisher exact probability method of the four grid table (*p* = 0.631 > 0.05; Table 3).

**Table 3:** Comparison of the effectiveness.

**Group**	**Effective**	**Relapse**	**Sum**	**Effective rate (%)**
Laser group	25	3	28	89.29
TUR group	27	3	30	90
Sum	52	6	58	89.66

## Discussion

In 1761, Morgagni et al. first proposed glandular cystitis a bladder mucosa dysplasia caused by a variety of factors (5). Glandular cystitis usually affects more women than men (6). The bladder triangle and bladder neck are regarded as the focal points of urodynamics, and their positions are relatively fixed. In addition, the bladder wall has no submucosa and lacks stretchability. Therefore, glandular cystitis is more likely to occur in the bladder triangle. Clinically, cystitis glandularis usually manifests as frequent urination, urgency, dysuria, hematuria, dysuria, pain in the suprapubic area and perineum, etc. And these symptoms are prone to recurring. In severe cases, acute urinary retention mayoccur (7). For the treatment of glandular cystitis, some people advocate conservative treatment such as the use of quinolone antibiotics and cyclooxygenase-2 inhibitors (3). Clinically, many patients with glandular cystitis have symptoms of overactive bladder. For such patients, measures can be taken to improve those symptoms. Highly selective M3 receptor blockers inhibit involuntary detrusor contractions to delayurination, having obvious effect in the treatment of symptoms of bladder over-activity (8–10).

There is a view that CG precancerous lesions, should be actively treated surgically. Currently, main treatment for glandular cystitis is transurethral resection. Among ways of it, transurethral electrocision of bladder lesion is the earliest surgical approach, and has been adopted as the most commononedue toits good results in the treatment of glandular cystitis, such as fewer traumas, shorter operation time, and faster patient recovery. However, this type of surgery has some shortcomings. During the treatment process, complications are more likely to occur, such as relatively more bleeding and post-operative bleeding, resulting in clot packing in the bladder, following too much hypertonic flushing fluid leading to dilution hyponatremia. Furthermore, it is easy to cause obturator nerve reflex during this operation process and cause bladder perforation (11, 12). The output center wavelength of the 2 μm laser ranges from 1,900 to 2,040 nm, and the maximum absorption peak of water to the laser is 1,940 nm. The process of absorption to 2 μm laser in the human tissue can produce a strong thermal effect, so as to achieve the effect of vaporization and cutting of the tissue (13, 14). Advantages of the use of 2 μm laser during operation areas follows: firstly, the combination process of 2 μm laser with water can produce efficient thermal effect for tissue cutting, vaporization and coagulation during surgery; next, the penetration depth of the 2 μm laser to tissue is only 0.2 mm, achieving precise operation during the operation and thus reduces surgical complications; finally, the use of 2 μm laser will not generate an electric field, avoiding the occurrence of obturator nerve reflex, moreover, it is also a suitable choice for high-risk patients carrying with cardiac pacemakers or cardiac stents to treat glandular cystitis surgically (13, 15).

It is these advantages mentioned above, that makes 2 μm laser in urology been widely used. We have summarized the following characteristics of clinical application of 2 μm laser to treat bladder mucosal: firstly, due to 2 μm laser performing precise cutting, the application of 2 μm laser can perform fine layered vaporization resection of the bladder mucosa and muscle layer; besides, due to its effective and rapid tissue cutting, vaporization and coagulation effects, the intraoperative operation is easy to control with clear layers of tissue and small amount of intraoperative blood loss, and the bladder blood clot packing has never occurred after the operation; next, given that the 2 μm laser penetration depth in the tissue is only 0.2 mm, bladder perforation has never occurred in the case of surgeon accurately distinguishing the bladder mucosa with muscle layer of the bladder; finally, because the 2 μm laser does not generate an electric field like an electric resection, all the transurethral 2 μm laser bladder lesion mucosal en bloc resection performed in our hospital have never caused obdurate nerve reflexes, which greatly improves the safety of the operation.

## Conclusion

In summary, transurethral 2 μm laser bladder lesion mucosal en bloc resection in the treatment of glandular cystitis has the advantages of precise cutting, clear layers of tissue structure, less bleeding, fewer complications, shorter operation time, and faster patient recovery. Therefore, transurethral 2 μm laser bladder lesion mucosal en bloc resection for the treatment of glandular cystitis is worthy of further clinical application. It needs to be further improved and developed in the future extensive clinical practice.

## Data Availability Statement

The original contributions presented in the study are included in the article/supplementary material, further inquiries can be directed to the corresponding author.

## Ethics Statement

The studies involving human participants were reviewed and approved by The Second Affiliated Hospital of Chifeng College of Science and Technology. The patients/participants provided their written informed consent to participate in this study.

## Author Contributions

CZ wrote this manuscript and analyzed the data. CM, YX, CZ, and KW collected the data. HX designed the study. All authors approved the submitted version.

## Conflict of Interest

The authors declare that the research was conducted in the absence of any commercial or financial relationships that could be construed as a potential conflict of interest.

## Publisher's Note

All claims expressed in this article are solely those of the authors and do not necessarily represent those of their affiliated organizations, or those of the publisher, the editors and the reviewers. Any product that may be evaluated in this article, or claim that may be made by its manufacturer, is not guaranteed or endorsed by the publisher.
